# Early Stage Oropharyngeal Carcinomas: Comparing Quality of Life for Different Treatment Modalities

**DOI:** 10.1155/2014/421964

**Published:** 2014-02-25

**Authors:** Don-Felix Ryzek, Konstantinos Mantsopoulos, Julian Künzel, Philipp Grundtner, Johannes Zenk, Heinrich Iro, Georgios Psychogios

**Affiliations:** Department of Otorhinolaryngology, Head and Neck Surgery, Friedrich Alexander University of Erlangen-Nuremberg, Waldstraße 1, 91054 Erlangen, Germany

## Abstract

*Objective*. To compare long-term quality of life outcomes after treating early stage oropharyngeal carcinoma either with surgery, surgery combined with radiotherapy, or surgery combined with chemoradiotherapy. *Methods*. Questionnaire based method: 111 eligible patients agreed to fill out a quality of life questionnaire. *Results*. Of the 32 scales contained in the EORTC's combined QLQ-C30 and HN35, 11 scales show significantly better results for the surgery-only treatment group when compared to either surgery combined with radiotherapy or surgery combined with any type of adjuvant therapy. These eleven scales are role function (*P* = 0.019/0.008), social function (*P* = 0.01/0.034), nausea (*P* = 0.017/0.025), pain (*P* = 0.014/0.023), financial problems (*P* = 0.030/0.012), speech (*P* = 0.02/0.015), social eating (*P* = 0.003/<0.001), mouth opening (*P* = 0.033/0.016), sticky saliva (*P* = 0.001/<0.001), swallowing (*P* < 0.001/<0.001), and dry mouth (*P* < 0.001/0.001). *Conclusion*. Treatment of early stage oropharyngeal carcinoma with surgery alone has definite advantages over treatments including any form of adjuvant therapy when considering quality of life. Advantages manifest themselves especially in functional aspects of the head and neck realm; however general health aspects as well as psychosocial aspects show improvements as well. This study does not show any indication of QOL-related drawbacks of surgery-only treatment approaches.

## 1. Introduction

Oropharyngeal Carcinomas (OPCs) represent up to 3% of all new cancer diagnoses in the United States and are a commonly occurring cancer of the head and neck region [1, 2]. Usually, these OPCs are diagnosed in more advanced stages and have poor prognosis. However, when they are diagnosed early on (at stages T1 and T2), they have good five-year survival estimates [3, 4]. Upon diagnosis in their early stages, transoral tumor resection alone or in combination with adjuvant radiotherapy or chemoradiotherapy offers very good oncologic results [5, 6]. All of these treatment modalities have showed their effectiveness in increasing survival estimates [7–9]. Since oncologic results are excellent, impact on quality of life becomes important when choosing treatment modality for the individual patient [10].

The uniqueness of the oropharyngeal anatomical region stems from its functional importance in activities such as eating and speaking. In addition, its highly exposed nature allows potential aesthetic defects to have a strong stigmatizing effect. Given these key functional and social aspects, and the fact that OPC as well as the different treatment options can have severe effects on all of these factors, it should be of no surprise that the patients' subjective quality of life (QOL) could be severely affected on social, physical, and psychological levels.

Considering the potentially different effect on QOL stemming from methodically different but comparably effective treatment methods, expected QOL outcomes should be an important factor when choosing the appropriate therapeutic approach [11]. Publicly, nonsurgical treatment methods are perceived as less invasive and thus are often favored due to the assumption that there may be a better QOL outcome [12, 13]. There is little conclusive data to support this conclusion and this study aims to shed light with regard to this issue. Furthermore, the aim of this study is to compare the QOL in patients after transoral resection of a small OPC with or without adjuvant treatment, in order to better understand the impact of the various parts of a multimodal treatment (surgery, radiotherapy, and chemotherapy) in long-term QOL.

## 2. Methods

The Ethics Commission of the FAU Erlangen-Nürnberg reviewed and subsequently approved the intended patient selection process and the study protocol as well as the required patient consent form. In order to be considered for the study, patients had to meet the following inclusion criteria: (1) tumor was located within the oropharynx, (2) primary tumor was of early stage (pT1 or pT2, N0-2), (3) no distant metastasis were detected (M0), (4) tumor was successfully treated with a minimum tumor free interval of 18 months after treatment, and (5) tumor was not a relapse of a previously existing tumor. The different forms of treatment considered were surgery alone (OP) or a combination of surgery and radio- and/or chemotherapy (OPRT/OPRCT). Excluded were all patients with a recurrent disease. For the purpose of this study, OPCs are defined as all carcinomas falling under the following groups of the ICD-10: C01, C05 without C05.0, C09, and C10.

An existing database comprised of follow-up patients with treated primary oropharyngeal carcinoma combined with a thorough screening of patients coming in for routine posttreatment follow-up was used to identify 160 eligible candidates.

Patients' QOL was evaluated in detail using German-language versions of two standardized questionnaires from the European Organization for Research and Treatment of Cancer (EORTC), specifically the Core Module [14] (EORTC-QLQ-C30) and the Head and Neck Cancer Module [15] (EORTC-QLQ-H&N35). The Core Module is designed as an assessment tool for cancer patients. It evaluates a total of nine multi-item scales including five functional scales (physical, role, cognitive, emotional, and social) and three symptom scales (fatigue, pain, and nausea and vomiting) as well as a global health and QOL-scale. Additionally, a number of single-item symptom measures are included. This Core Module is supplemented by the Head and Neck Cancer Module consisting of six multi-item scales designed to capture issues associated with cancers of the specified region and their treatment (pain, swallowing, speech, social eating, social contacts, and sexuality). In both modules, achievable scores for each scale range from zero to one hundred. Higher scores represent higher response levels; that is, in functional scales, a high score implicates more positive outcomes while high scores in symptom scales are congruent with more negative outcomes. Statistical analysis of the acquired data was done according to EORTC-QLQ scoring instructions. The resulting, dichotomized and grouped scores were compared and checked for significance using chi-square independence tests or Fischer tests when necessary for OP versus OPRT as well as for OP versus patients who received surgery and any form of adjuvant therapy (OP + adj). The latter group consists of OPRT as well as OPRCT. The phi-coefficient was used as a measure of association for significant results.

Missing items within the EORTC-QLQ were a minor issue when reviewing the questionnaires. Seven questionnaires that were sent out for home answering were missing items 9–19 of EORTC-QLQ-H&N35 due to a systematic error. Since the omissions were caused by a systematic error and not by patients' refusal to answer certain questions (because they felt uncomfortable to do so for instance) considering these single items as lost should have no further impact on the statistical proceedings. These single missing items were considered lost and not processed further.

The use of adjuvant therapy in form of RT or RCT was decided in our tumor board. Irradiation typically included the primary tumor site and the involved side of the neck. Various changes in treatment protocols, as well as technical developments, have been noted over the years. Today, however, typical indications for RCT include the presence of positive surgical margins when further surgery was not feasible, advanced neck disease, and extracapsular tumor spread. Typical indications for adjuvant RT include close margins, solitary cervical metastasis, and infiltration of lymph vessels or nerves in permanent histology. Sometimes a combination of soft criteria such as poor differentiation, large tumor dimension, or large tumor depth can result in offering the patient an adjuvant RT.

The standard tumor follow-up protocol of the ENT department was used to examine patients' medical status. This consisted of a patient interview and inspecting the treated regions visually (with aid of scopes), by palpation and, in indicated circumstances, by ultrasound. Results that were relevant to this study were documented on a form separate from the patient questionnaire.

## 3. Results

Of all eligible candidates, 111 volunteered to participate in the study anonymously between June 2011 and June 2012. 85 participants were males and 26 females. 80 patients were seen in person while 31 individuals preferred to participate by answering the questionnaire via mail. 26 patients were treated with surgery as their only form of treatment (OP-Group) while 33 patients received additional radiotherapy (OPRT-Group) and 52 patients had additional radiotherapy as well as chemotherapy (OPRCT-Group). Treated tumor sizes in all three groups were comparable. Detailed patient demographics according to therapy group are presented in Table 1.

The results of the EORTC-QLQ-C30 and the EORTC-QLQ/H&N35 for treatment groups OP, OPRT, and OPRCT are shown in Tables 2 and 3, respectively.

Table 4 contains four questionnaire scales (2 from EORTC-QLQ-C30 and 2 from EORTC-QLQ-HN35). For each item comparisons are made with regard to the percentage of patients that report a full score versus the percentage of patients that report a less than maximum score. A score of “100” can be equated with an optimal outcome. Items “role function” as well as “social function” (marked with italic font) show that the percentage of patients without any loss of function in these aspects is significantly higher when compared to OPRT or OP + adj treatment groups.

Table 5 is comprised of twenty-two scales (EORTC-QLQ-C30: 9, EORTC-QLQ-HN35: 13). These scales are compared as to how many patients reported scores of zero versus how many patients reported scores above zero. For all items, a response of “0” represented a better outcome for the patient. Seven of these scales (EORTC-QLQ-C30: 3, EORTC-QLQ-HN35: 4) show significant correlation with *P* ≤ 0.05. For all seven scales (nausea, pain, financial problems, HN speech, HN social eating, HN opening mouth, and HN sticky saliva), a surgery-only approach yielded more patients who reported optimal results when compared to OPRT and OP + adj. Five items of the scale HN social eating are missing answers due to the aforementioned systematic error, however, only to a negligible extent such that there is no effect on the trend.

The six final items (EORTC-QLQ-C30: 3, EORTC-QLQ-HN35: 3) as well as the overall Quality of Life score are compared in Table 6. The table reflects how many patients of each treatment group reported above- versus below-average outcomes. Notably, higher scores in the EORTC-QLQ-C30 items represent better outcomes, whereas lower scores in EORTC-QLQ-HN35 items are better. Items “HN swallowing” and “HN dry mouth” have significant results (*P* ≤ 0.05) illustrating that an above-average number of members of the surgery-only treatment group reported below-average scores, that is, above-average outcomes.

## 4. Discussion

Quality of life (QOL) is a complex, multifaceted construct that is challenging to accurately measure. With the advent of tools, such as the EORTC's set of QOL-questionnaires some two decades ago, the possibility of comparing medically influenced outcomes on QOL allowed researchers to look beyond survival as the sole measure of successful medical intervention. This becomes especially valuable when faced with medical interventions that generate similar survival estimates and may primarily differ in terms of patients' perceived QOL. Currently, there are few studies comparing QOL outcomes in different treatment modalities of OPC [11, 13]. Many of these focus on the aftermath of advanced OPC while little research about early stage OPCs has surfaced [12]. Tschiesner et al. and Mowry et al. both come to the conclusion that treatments involving surgery have more desirable QOL-outcomes for advanced stage OPCs [4, 16]. In this particular study, long-term outcomes of different treatment modalities for early stage OPC are considered the deciding factor when choosing a course of treatment for patients.

As shown in previous studies, individuals with limited OPC make up a group of patients with excellent oncologic result [5, 17]. A transoral resection of the tumor is possible in most cases. The development of laser microsurgery and transoral robotic surgery improves the effectiveness of surgical treatment [18]. In a recently published study we were able to show that in the absence of certain prognostic factors such as tumor dimension of less than 2 cm and tumor depth of less than 5 mm surgery as a single modality treatment offers very good results in patients with pT1-2N0-1 OPC [3]. Another important aspect is the role of HPV infection in OPC. Since these patients are usually younger and have a better prognosis, desintensification of treatment is currently being discussed for this patient group [19, 20]. Therefore data on QOL of these patients compared to patients treated with surgery and adjuvant radio(chemo)therapy could play an important role in choosing the optimal treatment modality [11]. We hypothesized that the expected improvements in reported QOL when choosing surgery as the sole treatment option were due to not exposing patients to the side effects that may have been a consequence of additional treatments with radio- and/or chemotherapy. Furthermore this therapeutic tool could be preserved for cases of recurrence of second primary tumors, which are known to occur in up to 25% of patients. In particular, this study was aimed to look at surgery-only treatment versus two distinct groups: surgery combined with radiotherapy alone and surgery plus adjuvant therapies in general. For the purpose of this study, adjuvant therapies were either radiotherapy alone or in combination with chemotherapy.

Regardless of treatment modality, most patients report acceptable QOL outcomes. As is expected, the vast majority of complaints that do exist from long-term survivors of head and neck cancers focus on that specific region. Funk et al. identified that the functional aspects of eating and swallowing were most impacted by the disease [21]. The trend of these findings is supported by this study as well [22]. Patients treated with adjuvant therapies in particular show a similar complaint profile that highlights loss of oral functions. It is notable however that a large majority of OP-only patients do not express any complaints regarding the head and neck region in this particular sample set.

**Figure 1:**
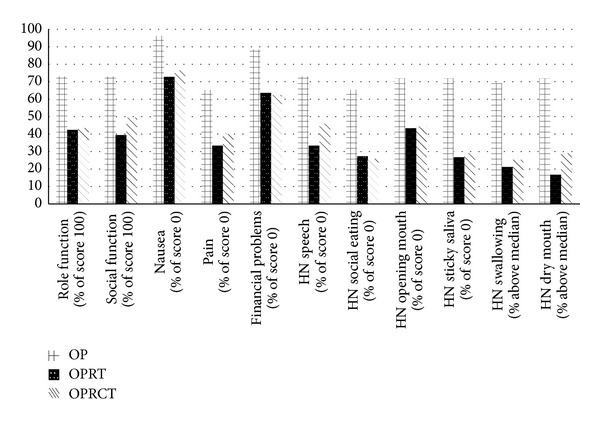
Statistically significant results.

Using the general and H&N specific QOL-questionnaires of the EORTC to facilitate comparison between outcomes showed us statistically significant differential outcomes within nine of the measured scales, as illustrated in Figure 1. Six of these scales (HN speech, HN social eating, HN opening mouth, HN sticky saliva, HN swallowing, and HN dry mouth) are specific to the head and neck region while three (pain, nausea, and financial problems) are identified with the general section of the QOL-questionnaire. Scores in all nine mentioned scales favor surgery-only treatment as having the better outcome. The results for the physical function scale are less conclusive, but there is a clear trend towards significance.

Apart from a small cohort size, the main limitation of this study is the existence of numerous confounding factors, which cannot be eliminated. Treatment modality is only one variable that is responsible for patients' perceived QOL. Other factors that have been identified to impact QOL of OPC survivors are age, gender, marital status, comorbidities, malnutrition, and staging of the tumor [23]. These problems should be addressed with a prospective follow-up study with a larger cohort size.

## 5. Conclusion

Regardless of the treatment modality chosen for early stage OPC, overall quality of life as determined by “Global Health Status” of the QLQ-C30 can be considered good. Nonetheless, it becomes apparent that treatment via surgery alone has definite advantages over treatments including any form of adjuvant therapy. Even though these advantages manifest themselves especially in functional aspects of the head and neck realm, they are not exclusive to this region. General health aspects as well as psychosocial functions seem to be improved as well. In this study, there are no hints of a potential QOL-related drawback of surgery-only treatment approaches.

A continuation of this study to achieve larger sample sizes over the coming year is advisable in order to corroborate above findings and potentially discover further statistically significant differences between the treatment methods named.

## Figures and Tables

**Table 1:** Detailed description of demographics, treatment modalities, and histological differentiation according to therapy.

Characteristics	OP	OPRT	OPRCT
Gender	Male: 20 (76.9%) Female: 6 (23.1%)	Male: 24 (70.6%) Female: 10 (29.4%)	Male: 42 (82.4%) Female: 9 (17.6%)

Age (years)	Median: 55 Range: 40–69	Median: 54 Range: 37–78	Median: 54 Range: 36–73

Smoking	Smokers: 12 (15.4%), Nonsmokers: 4 (32.7%) n.a.: 10 (38.5%)	Smokers: 13 (38.2%), Nonsmokers: 3 (8.8%) n.a.: 18 (49%)	Smokers: 18 (35.3%), Nonsmokers: 4 (7.8%) n.a.: 29 (56.8%)

Follow-up (years)	Median: 2.99 Range: 0.28–10.69	Median: 4.44 Range: 0.93–15.85	Median: 4.77 Range: 0.22–13.05

Localization	Base of the tongue: 5 (19.2%) Palatine tonsil: 9 (34.6%) Palatal arch 11 (42.3%) Oropharynx NOS: 1 (3.8%)	Base of the tongue: 8 (23.5%) Palatine tonsil: 18 (52.9%) Palatal arch 5 (14.7%) Oropharynx NOS: 3 (8.8%)	Base of the tongue: 13 (25.5%) Palatine tonsil: 31 (60.8%) Palatal arch 2 (3.9%) Oropharynx NOS: 5 (9.8%)

T-category	pT1: 17 (65.3%) pT2: 9 (34.6%)	pT1: 15 (44.1%) pT2: 19 (55.9%)	pT1: 21 (41.2%) pT2: 30 (58.8%)

Tumor-depth (mm)*	Median: 4 Range: 1–35	Median: 5 Range: 2–25	Median: 5 Range: 1–19

Tumor-size (mm)	Median: 21 Range: 6–37	Median: 22 Range: 5–40	Median: 22 Range: 7–38

N-category (pooled)	0: 69.2% 1: 19.2% 2: 11.5%	0: 44.1% 1: 20.6% 2: 35.3%	0: 9.8% 1: 23.5% 2: 66.7%

Neck dissection	Yes: 20 (76.9%) No: 6 (23.1%)	Yes: 34 (100%)	Yes: 51 (100%)

Surgical technique	TLM: 3 (11.5%) Electrocautery: 33 (89.5%)	TLM: 6 (17.6%) Electrocautery: 27 (79.5%) Combined: 1 (2.9%)	TLM: 10 (19.6%) Electrocautery: 40 (78.4%) Combined: 1 (2.0%)

Histological differentiation	G2: 20 (76.9%) G3: 6 (23.1%)	G1: 1 (2.9%) G2: 20 (61.8%) G3: 6 (35.3%)	G2: 27 (52.9%) G3: 24 (47.1%)

*Tumor-depth is a calculated function of tumor extent and depth of infiltration.

TLM: transoral laser microsurgery, RT: radiotherapy, and RCT: radiochemotherapy.

**Table 2:** EORTC-QLQ-C30 scores for all three treatment groups.

Item	OP	OPRT	OPRCT
	Median (95%-confidence interval of mean)	Median (95%-confidence interval of mean)	Median (95%-confidence interval of mean)
Global health status	75.00 (62.79–80.16)	66.67 (56.85–72.95)	66.67 (59.22–70.91)
Functional scales			
Physical	100 (87.33–97.28)	86.67 (75.34–88.30)	93.33 (79.68–91.66)
Role	100 (85.28–98.05)	83.33 (58.70–82.71)	83.33 (64.70–81.46)
Emotional	83.33 (72.59–88.95)	75.00 (59.79–80.79)	83.33 (66.66–80.99)
Cognitive	100 (80.90–96.02)	83.33 (68.77–87.80)	100 (76.23–89.79)
Social	100 (78.80–98.13)	83.33 (64.59–85.92)	100 (71.26–86.43)
Symptom scales			
Fatigue	16.67 (13.25–31.20)	22.22 (20.16–41.45)	27.78 (22.09–39.02)
Nausea and vomiting	0 (−0.68–1.96)	0 (2.39–11.75)	0 (1.78–9.76)
Pain	0 (5.16–21.77)	16.67 (18.31–39.26)	16.67 (17.18–34.75)
Dyspnea	0 (9.79–33.80)	0 (8.53–29.86)	0 (11.15–27.31)
Insomnia	0 (14.64–41.77)	0 (11.24–35.23)	0 (16.52–37.33)
Appetite loss	0 (−1.08–16.47)	0 (8.12–30.26)	0 (8.68–27.22)
Constipation	0 (2.26–20.82)	0 (5.68–28.66)	0 (3.16–16.07)
Diarrhea	0 (1.94–18.57)	0 (2.15–14.01)	0 (1.37–10.16)
Financial problems	0 (−2.17–20.11)	0 (7.74–24.58)	0 (12.26–28.76)

**Table 3:** EORTC-QLQ-H&N35 scores for all three treatment groups.

Item	OP	OPRT	OPRCT
	Median (95%-confidence interval of mean)	Median (95%-confidence interval of mean)	Median (95%-confidence interval of mean)
HN pain	0 (3.37–13.97)	8.33 (8.37–24.39)	8.33 (12.40–24.76)
HN swallowing	0 (3.78–20.88)	25.00 (19.34–32.39)	20.83 (18.82–29.44)
HN senses	0 (2.72–15.94)	0 (8.15–29.78)	0 (11.69–27.20)
HN speech	0 (0.58–16.31)	11.11 (11.67–28.18)	0 (9.39–22.56)
HN social eating	0 (2.25–22.42)	25.00 (16.18–37.65)	20.833 (18.97–35.20)
HN social contact	0 (0.48–6.45)	0 (3.21–19.32)	0 (4.21–13.29)
HN sexuality	0 (8.08–31.92)	0 (12.14–39.58)	0 (17.08–37.78)
HN teeth	0 (6.54–36.13)	0 (16.11–48.25)	0 (17.59–40.74)
HN opening mouth	0 (5.68–34.32)	33.33 (22.67–50.89)	33.33 (25.58–46.64)
HN dry mouth	0 (14.30–44.36)	100 (66.78–89.54)	66.67 (49.93–72.29)
HN sticky saliva	0 (3.60–23.06)	66.67 (37.11–66.33)	66.67 (37.50–59.72)
HN coughed	0 (12.41–35.59)	33.33 (16.65–40.82)	33.33 (23.38–41.89)
HN felt ill	0 (3.03–15.64)	0 (1.71–23.58)	0 (8.20–25.14)
HN pain killers	0 (19.36–60.64)	0 (10.28–44.89)	0 (17.65–44.85)
HN nutritional supplements	0 (−4.26–12.26)	0 (7.57–40.70)	0 (5.73–27.60)
HN feeding tube	0 (−1.69–25.69)	0 (−2.91–16.71)	0 (0.22–16.44)
HN weight loss	0 (6.01–41.99)	0 (0.44–27.14)	0 (2.80–22.20)
HN weight gain	0 (9.08–46.92)	0 (0.44–27.14)	0 (−0.85–13.35)

**Table 4:** Comparing OP with OPRT and OP + adj—scale group 1 (percent within therapy group).

Item	OP		OPRT		OP + adj		OP versus OPRT *P*/Phi	OP versus OP + adj *P*/Phi
	Score 100	>100	100	>100	100	>100		
Role function	*73.1 *	*26.9 *	*42.4 *	*57.6 *	*43.5 *	*56.5 *	*0.019/0.268 *	*0.008/0.250 *
Social function	*73.1 *	*26.9 *	*39.4 *	*60.6 *	*49.4 *	*50.6 *	*0.01/0.336 *	*0.034/0.201 *
HN sexuality	61.5	38.5	51.5	48.5	54.1	45.9	NS	NS
HN nutritional supplements	*96.2 *	*3.8 *	*76.7 *	*23.3 *	80.0	20.0	*0.038/0.278 *	NS

NS: Results are statistically not significant.

**Table 5:** Comparing OP with OPRT and OP + adj—scale group 2 (percent within therapy group).

Item	OP		OPRT		OP + adj		OP versus OPRT *P*/Phi	OP versus OP + adj *P*/Phi
	Score 0	>0	0	>0	0	>0		
Fatigue	30.8	69.2	30.3	69.7	32.9	67.1	NS	NS
Nausea	*96.2 *	*3.8 *	*72.7 *	*27.3 *	*76.5 *	*23.5 *	*0.017/0.310 *	*0.025/0.213 *
Pain	*65.4 *	*34.6 *	*33.3 *	*66.7 *	*40.0 *	*60.0 *	*0.014/0.319 *	*0.023/0.216 *
Dyspnea	57.7	42.3	66.7	33.3	64.7	35.3	NS	NS
Insomnia	53.8	46.2	60.0	40.0	60.0	40.0	NS	NS
Appetite loss	84.6	15.4	69.7	30.3	71.8	28.2	NS	NS
Constipation	76.9	23.1	72.7	27.3	78.8	21.2	NS	NS
Diarrhea	76.9	23.1	78.8	21.2	83.5	16.5	NS	NS
Financial problems	*88.5 *	*11.5 *	*63.6 *	*36.4 *	*62.4 *	*37.6 *	*0.030/0.283 *	*0.012/0.238 *
HN pain	53.8	46.2	42.4	57.6	40.0	60.0	NS	NS
HN speech	*73.1 *	*26.9 *	*33.3 *	*66.7 *	*45.9 *	*54.1 *	*0.002/0.395 *	*0.015/0.231 *
HN social eating	*65.4 *	*34.6 *	*27.3 *	*72.7 *	*25.9 *	*74.1 *	*0.003/0.381 *	*<0.001/0.350 *
HN social contact	76.9	23.1	51.5	48.2	60.0	40.0	*0.045/0.261 *	NS
HN teeth*	68.0	32.0	53.3	46.7	57.0	43.0	NS	NS
HN opening mouth*	*72.0 *	*28.0 *	*43.3 *	*56.7 *	*44.3 *	*55.7 *	*0.033/0.288 *	*0.016/0.237 *
HN sticky saliva*	*72.0 *	*28.0 *	*26.7 *	*73.3 *	*29.1 *	*70.9 *	*0.001/0.470 *	*<0.001/0.375 *
HN coughed*	52.0	48.0	50.0	50.0	*41.8 *	*58.2 *	NS	NS
HN felt ill*	72.0	28.0	80.0	20.0	73.4	26.6	NS	NS
HN pain killers	61.5	38.5	66.7	33.3	54.1	45.9	NS	NS
HN feeding tube	88.5	11.5	90.0	10.0	91.8	8.2	NS	NS
HN weight loss	76.9	23.1	83.3	16.7	85.9	14.1	NS	NS
HN weight gain	*73.1 *	*26.9 *	83.3	16.7	*89.4 *	*10.6 *	NS	*0.045/−0.200 *

*Items were calculated with a total of 7 missings. This is due to a systematic error described more closely in Materials and Methods section.

NS: results are statistically not significant.

**Table 6:** Comparing OP with OPRT and OP + adj—scale group 3 (percent within therapy group).

Item	Median score	OP		OPRT		OP + adj		OP versus OPRT *P*/Phi	OP versus OP + adj *P*/Phi
		<Median	≥Median	<Median	≥Median	<Median	≥Median		
Overall QOL	66.667	19.2	80.8	27.3	72.7	35.3	64.7	NS	NS
Physical function	93.33	*26.9 *	*73.1 *	*63.6 *	*36.4 *	48.2	51.8	*0.005/−0.307 *	NS
Emotional function	83.33	30.8	69.2	51.5	48.5	48.2	51.8	NS	NS
Cognitive function	83.33	15.4	84.6	30.3	69.7	27.1	72.9	NS	NS
HN swallowing	*16.667 *	*69.2 *	*30.8 *	*21.2 *	*78.8 *	*25.3 *	*74.7 *	*<0.001/0.482 *	*<0.001/0.398 *
HN senses	12.5	72.0	28	53.6	46.4	54.4	45.6	NS	NS
HN dry mouth	*66.67 *	*72.0 *	*28 *	*16.7 *	*83.3 *	*29.1 *	*70.9 *	*<0.001/0.559 *	*<0.001/0.375 *

NS: results are statistically not significant.
